# Potential effect of chloroquine and propranolol combination to treat colorectal and triple-negative breast cancers

**DOI:** 10.1038/s41598-023-34793-6

**Published:** 2023-05-16

**Authors:** L. E. Anselmino, M. V. Baglioni, G. Reynoso, V. R. Rozados, O. G. Scharovsky, M. J. Rico, M. Menacho-Márquez

**Affiliations:** 1 Instituto de Inmunología Clínica y Experimental de Rosario (IDICER, CONICET-UNR), Facultad de Ciencias Médicas (UNR), 3100 Rosario, Santa Fe Argentina; 2 CONICET, Rosario, Argentina; 3 Instituto de Genética Experimental, Facultad de Ciencias Médicas, 3100 Rosario, Santa Fe Argentina; 4 Centro de Investigación y Producción de Reactivos Biológicos (CIPReB), Facultad de Ciencias Médicas, Suipacha, 660, Rosario, Argentina

**Keywords:** Cancer, Breast cancer, Cancer therapy, Gastrointestinal cancer

## Abstract

Drug repositioning explores the reuse of non-cancer drugs to treat tumors. In this work, we evaluated the effect of the combination of chloroquine and propranolol on colorectal and triple-negative breast cancers. Using as in vitro models the colorectal cancer cell lines HCT116, HT29, and CT26, and as triple-negative breast cancer models the 4T1, M-406, and MDA-MB-231 cell lines, we evaluated the effect of the drugs combination on the viability, apoptosis, clonogenicity, and cellular migratory capacity. To explore the in vivo effects of the combination on tumor growth and metastasis development we employed graft models in BALB/c, nude, and CBi mice. In vitro studies showed that combined treatment decreased cell viability in a dose-dependent manner and increased apoptosis. Also, we demonstrated that these drugs act synergically and that it affects clonogenicity and migration. In vivo studies indicated that this drug combination was effective on colorectal models but only partially on breast cancer. These results contributed to the search for new and safe treatments for colorectal and triple-negative carcinomas.

## Introduction

Several diseases share cell signaling pathways and targets. On the other hand, a high proportion of the drugs available in the market have more than one target. These considerations and some epidemiologic studies led to drug repurposing in oncology an interesting therapeutic proposal for cancer treatment that has emerged in the last decades. It consists of the use of already known drugs, which were designed and are utilized for other pathologies, to be used for the treatment of malignant tumors. This strategy uses known drugs, already employed in the clinics, reducing the risks associated with toxicity and lowering the costs of therapies^1^.

Although the development of chemotherapies in clinical oncology began with the use of individual agents that demonstrated antiproliferative properties, it is now known that anticancer drugs are often more effective when used in combination.

Chloroquine (CQ) is a well-tolerated antimalarial drug that is also used as an anti-inflammatory. It can interfere with DNA synthesis and repair and it is also able to inhibit autophagy^2^. Propranolol (P) is a β-blocker mainly used to treat hypertension. Several studies have shown the usefulness of these drugs as sole agents for the treatment of different types of cancer^3^, however the effect of their combination was not yet evaluated.

In this context, we aimed to explore the antitumor effect of CQ + P combination on in vitro and in vivo models of triple-negative breast cancer (TNBC) and colorectal cancer (CRC), two of the more prevalent cancer types worldwide.

## Materials and methods

### Cell culture

To carry out our experiments in breast cancer models we used three triple-negative (Estrogen, Progesterone and HER2/neu negative receptors) mammary tumor cells: mouse 4T1 and M-406 cells (the latter obtained as described before^4^), and human MDA-MB-231 cells.

As models for CRC, we used three cell lines: CT26 derived from a female BALB/c mouse colorectal carcinoma, and HCT116 and HT29 obtained from male human colon adenocarcinomas.

Canine kidney MDCK cells were used as non-tumor control cells.

Cells were incubated at 37 °C with 5% CO_2_ and cultured in DMEM (MDA-MB-231, HT29, HCT116 and MDCK) or RPMI (4T1, M-406 and CT26) media supplemented with 10% fetal calf serum, penicillin (10 μg/ml) and streptomycin (100 μg/ml). Cells were periodically tested for mycoplasma contamination.

### Drugs

Propranolol (Propranolol hydrochloride; Sigma Aldrich) and chloroquine (Chloroquine hydrochloride; Sigma Aldrich) were dissolved in distilled water and stored at − 20 °C.

### Proliferation assays

For assessment of cell proliferation inhibition by treatments, 2.5 × 10^3^ cells/well were plated on 96-well culture plates. After attachment, different concentrations of the individual or combined drugs were added. After 36 h of incubation, the number of living cells was estimated indirectly by the tetrazolium salts reduction method (MTT, Sigma Aldrich) as previously described^5^. Viability was expressed as the percentage of control untreated cells.

Half maximal inhibitory concentration (IC50) values were obtained from the absorbance curves as a function of the control cells by using the ED50plus v1.0 program.

### Clonogenic assay

Cells (500/well) were plated on 6-well plates. After attachment, the TNBC cells were cultured in the presence of 1 μM CQ and/or 1 μM P for 6 days, while CRC cells were cultured in the presence of 2.5 μM CQ and/or 2.5 μM P for 8 days. Fresh medium and drug were renewed every 48 h. Photos of the clones were taken at different times and their size was estimated by measuring colony diameters with the ImageJ software (version 1.53t, https://imagej.nih.gov/ij). After fixing the cells with 4% paraformaldehyde (PFA), colonies were stained with Giemsa to allow quantification.

### Apoptosis

Cells were plated on 6-well plates and when they reached 70% confluence, CQ and/or P were added at the indicated concentrations. After 24 h cells were collected, washed and stained with Annexin V-FITC (AP-Biotech) and Propidium Iodide. Apoptotic rates (Positive Annexin V-FITC cells) were determined by flow cytometry (Becton Dickinson FACS Aria II).

### Migration

Cells were plated until subconfluency. Serum content was reduced to 0.1%. Wounds were made on the cell monolayer with a tip and treatments were added and maintained under these conditions during the whole assay. Cell migration was estimated by measuring the closure of the initial wound in non-proliferative conditions. Pictures were taken at the indicated times. Quantification was performed using the ImageJ software.

### Animal studies

Adult 8-week-old female BALB/c and nude mice (N:NIH(S)-*Fox1*^nu^) were obtained from the School of Veterinary Sciences at the National University of La Plata. Adult 8-week-old female CBi mice were obtained from our breeding facilities. Mice were treated in accordance with the Canadian Council on Animal Care and ARRIVE 2.0 guidelines. Animals were fed with commercial chow and water ad libitum, and maintained in a 12 h light/dark cycle at the CIPReB facilities (Centro de Investigación y Producción de Reactivos Biológicos, Medicine School, National University of Rosario). For all experiments, animals (N = 5–6/group) were distributed and treated as follows: Control: regular drinking water; CQ: chloroquine in drinking water (70 mg/kg BW/day); P: propranolol in drinking water (7 mg/kg BW/day); CQ + P: chloroquine and propranolol treatments combined.

All experimental protocols involving animals were approved by the “Committee for the Care and Use of Laboratory Animals (CICUAL)” of the Rosario Medical School.

### Graft models

2 × 10^6^ HCT116 or 5 × 10^5^ CT26 viable cells were resuspended in PBS (100 μl) and injected subcutaneously in a group of nude or BALB/c mice. 5 × 10^3^ viable 4T1 cells were resuspended in PBS (100 μl) and injected orthotopically into the fourth right mammary gland of BALB/c female mice. For M-406 tumorigenesis, a tumor fragment of 1 mm^3^ was orthotopically implanted in the fat pad, in the right mammary flank of CBi mice. At the indicated time, mice were randomly distributed in groups and treated as described before. Tumor volume (V) was estimated by measuring tumor length (a) and width (b) with a caliper, with the formula V = 0.4ab^2^. The body weight of the mice was recorded during the whole experiment. After sacrifice, tumors, spleens, livers and lungs were extracted, fixed in 4% formaldehyde for 24 h and embedded in paraffin for standard hematoxylin/eosin staining. Metastasis were identified by visualization of stained samples under a microscope (Olympus BX40).

### Statistics

Single comparisons between two groups were performed with the Student's t-test, multiple comparisons were carried out using ANOVA and Tukey–Kramer multiple comparison tests to examine differences between groups. Data obtained were analyzed using with GraphPad Software Prism version 8, San Diego, CA USA (www.graphpad.com). Unless indicated, data are expressed as mean ± SEM and are representative of three independent biological replicates (n = 3). P values equal to or lower than 0.05 were considered statistically significant.

### Animal statements

Authors declare that all experimental protocols involving animals were approved by the “Committee for the Care and Use of Laboratory Animals (CICUAL)” of the Rosario Medical School. Mice were obtained from the School of Veterinary Sciences at the National University of La Plata and treated in accordance with the Canadian Council on Animal Care and ARRIVE guidelines. Animals were fed with commercial chow and water ad libitum, and maintained in a 12 h light/dark cycle at the CIPReB facilities (Centro de Investigación y Producción de Reactivos Biológicos, Medicine School, National University of Rosario).

## Results

**Figure 1:**
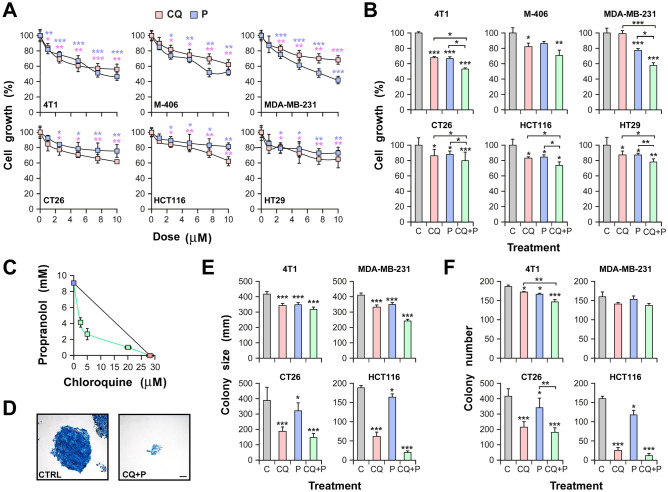
Chloroquine and propranolol affect the growth of TNBC and CRC cells. (**A**) TNBC (up) and CRC (bottom) cells were incubated with the indicated doses of CQ or P for 36 h. The number of living cells was estimated by the tetrazolium salts reduction method. Significant differences between treatment and control were evaluated with Student's t-test (**B**) Cell growth was estimated as before in control untreated cells (C) and cells treated with CQ, P and its combination (CQ + P) for 36 h. Up: TNBC cells were treated with 1 µM CQ and 1 µM P; Bottom: CRC cells were treated with 2.5 µM CQ and 2.5 µM P. (**C**) Isobologram for 4T1 cells: light blue and light red squares indicate IC50 values for each drug; green squares indicate the IC50 value for P in presence of an established concentration of CQ, and the IC50 value for CQ in presence of an established concentration of P. (**D**) Representative picture of HCT116 colonies. (**E, F**) 500 cells/well were cultured with or without treatment during 6–8 days. Colonies were stained with Giemsa to allow their quantification and colony size was estimated with ImageJ software. Scale bar = 100 µm. n = 3 for all experiments presented. Significant differences between groups were evaluated with ANOVA and Tukey–Kramer multiple comparison tests. *(P > 0.05); **(P > 0.01); ***(P > 0.001).

The effect on cell growth of the repurposed drugs CQ and P was evaluated at increasing concentrations for the specified cell lines. The serial intervals tested for each drug were chosen based on previous studies by our research group for propranolol^6^ and based on the literature for chloroquine^7^. Both drugs reduced cell growth in breast and colorectal cancer cell lines in a dose dependent manner (Fig. 1A). According to these results, we chose doses lower than IC50 for both drugs that were effective in significantly reducing cell proliferation compared to control but not lethal. The effect of combined treatment at specific concentrations was determined (1 µM CQ and P for breast cells and 2.5 µM CQ and P for colorectal cells). From that moment on, those doses were used in the following experiments. Noteworthy, combined treatment with CQ + P showed a stronger effect on cell growth than individual treatments for all the cell lines tested (Fig. 1B).

In order to evaluate the type of interaction between both drugs, we developed an isobologram. For that purpose, 4T1 cells were incubated with different concentrations of each drug in the presence of the corresponding dose of the other and the IC50 was calculated (Fig. 1C). After plotting the additive curve, it was evident that the effect of CQ + P combination was stronger than the sum of individual effects, hence indicating that these two drugs act in a synergistic rather than in an additive manner. To assess overall cytotoxicity, we tested the effect of individual and combined treatments on the non-tumoral cell line MDCK observing no significant changes in terms of growth or proliferation (Supplementary Fig. S1A,B).

Considering that repurposed drugs such as CQ and P are usually administered in a metronomic schedule, we determined the effect of continuous exposure of cells to these drugs and compared the impact of continuous (144 h) versus short-term (24 h) treatment on 4T1 cells. IC50 values obtained under these conditions indicated that continuous treatment with CQ or P increased cell sensitivity (Table 1).

**Table 1:** IC50 values for CQ and P were estimated for short (24 h) and long (144 h) treatments.

Cell lines	Chloroquine		Propranolol	
	IC50 (µM) 24 h	IC50 (µM) 144 h	IC50 (µM) 24 h	IC50 (µM) 144 h
4T1	27.64 ± 2.43	4.89 ± 0.42***	9.1 ± 2.47	0.10 ± 0.015***
MDA-MB-231	30.48 ± 10.23	15.31 ± 6.19	7.91 ± 0.20	0.21 ± 0.01***

Results are expressed as mean ± SEM; ***P < 0.001.

To deepen the proliferation studies, we assessed the clonal expansion capacity of cells in the presence of CQ and/or P. As expected for their effect on proliferation, individual treatments partially affected colony potential, but the combination of drugs promoted a more drastic effect on colony size (Fig. 1D,E). Indeed, the clonogenic potential was also affected by CQ, P and their combination in CRC cells, while a mild effect was only observed for 4T1 TNBC cells (Fig. 1F).

**Figure 2:**
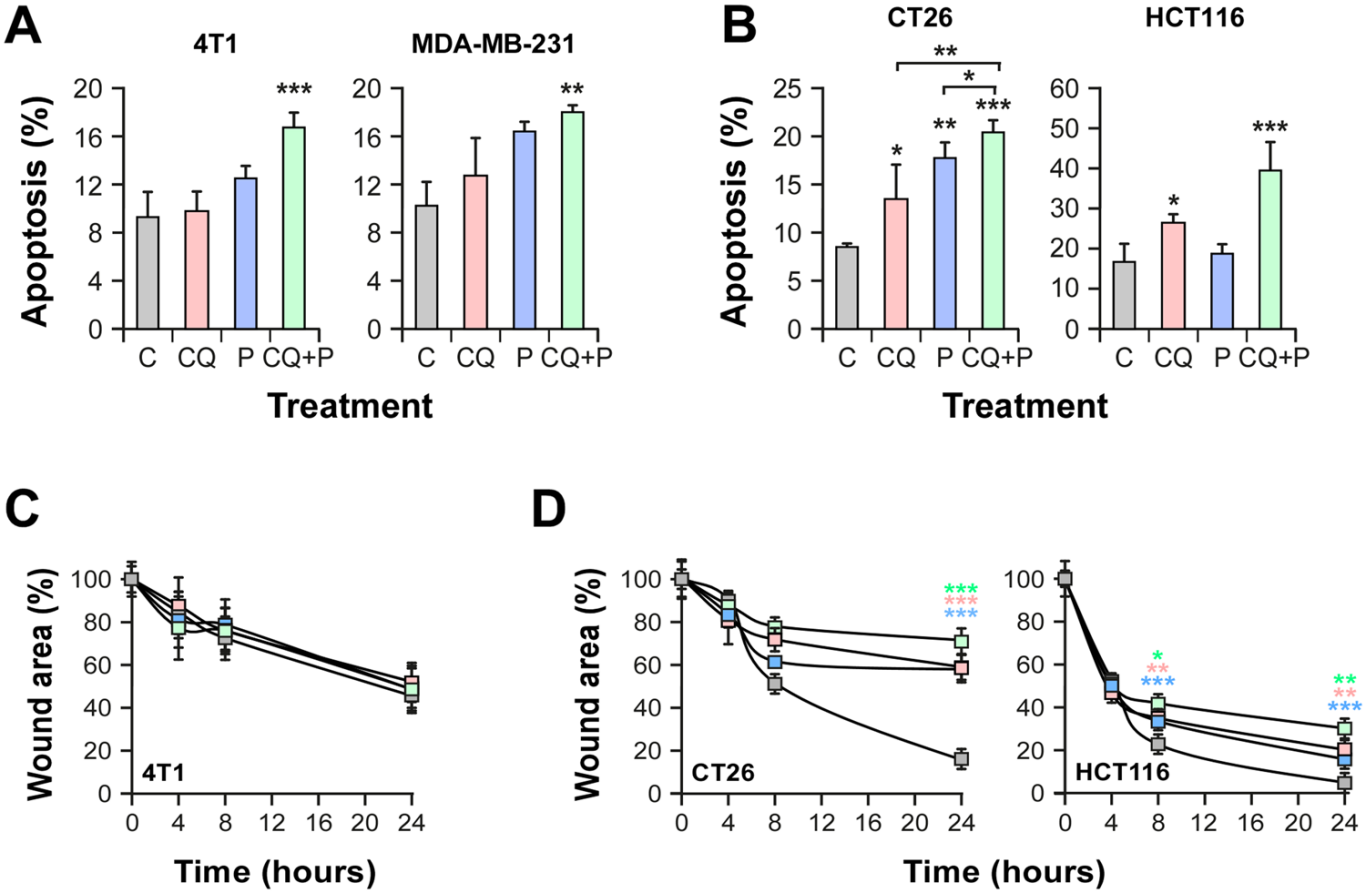
Combination of chloroquine and propranolol triggers apoptosis of TNBC and CRC cells and impairs migration of CRC cells. (**A**) TNBC cells were incubated with 1 µM CQ and/or 2.5 µM P (n = 3). (**B**) CRC cells were incubated with 2.5 µM CQ and/or 2.5 µM P (n = 3). Annexin V-FITC Propidium Iodide staining was used to quantify apoptotic cells via flow cytometry after 24 h of treatment. (**C**, **D**) The migratory ability of tumor cells was estimated by a wound-healing assay. Quantification of the healing process was performed using the ImageJ software. Significant differences between groups were evaluated with ANOVA and Tukey–Kramer multiple comparison tests: *(P < 0.05); **(P < 0.01); ***(P < 0.001).

Tumor cells have the ability to evade apoptotic signals. As consequence it is desirable for cancer therapy to induce the death of malignant cells. For that reason, we explored the potential of these treatments to trigger apoptosis by Annexin V^+^/IP staining. Interestingly, only combined treatment was capable to induce cell death significantly both in breast and CRC cells (Fig. 2A,B).

Metastasis are the main cause of death associated with cancer. During the development of metastases, malignant cells must set in motion several events to promote cell migration in order to colonize new tissues. Hence, we explored with a wound-healing assay, the effect of CQ and P, as individual or combined treatments, on the migratory capacity of tumor cells. Neither CQ and P, nor the combination, were able to prevent in vitro migration of TNBC cells (Fig. 2C). On the contrary, CQ, P and their combination were effective in preventing the migration of CRC cells (Fig. 2D). Interestingly, the combined treatment increased the inhibition of migration triggered by individual treatments.

**Figure 3:**
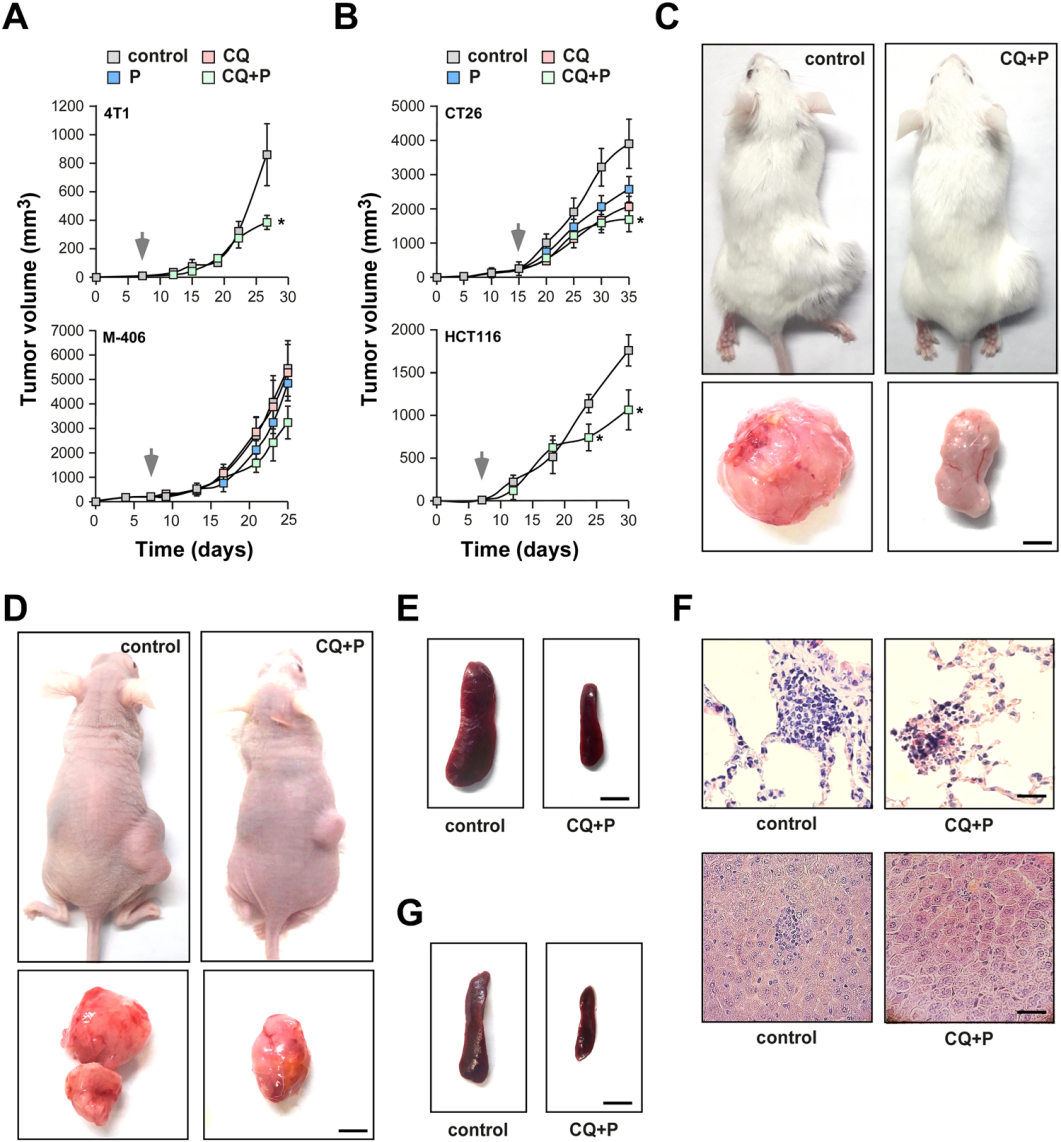
Effects of chloroquine and propranolol combination in vivo. BALB/c mice were subcutaneously challenged with 4T1 or CT26 cells (n = 6 per treatment group); immunodeficient mice were injected in the same way with HCT116 human cells (n = 5 per treatment group); an M-406 tumor fragment of around 1 mm^3^ was orthotopically implanted in the fat pad of the right mammary flank of CBi mice (n = 5 per treatment group). Gray arrows indicate the beginning of the treatment (CQ: 70 mg/kg BW/day; P: 7 mg/kg BW/day; CQ + P: combination in the same doses); in all cases tumor size was measured every three days with a caliper and tumor volume was estimated. (**A**) TNBC models, (**B**) CRC models. At the end of the experiment, animals were sacrificed for necropsy. (**C**, **E**) Representative pictures of BALB/c mice with CT26 tumors and spleens (scale bar = 0.5 mm). (**D**, **G**) Representative pictures of nude mice with HCT116 tumors and spleens (scale bar = 0.5 mm). (**F**) Representative pictures of sections of lungs (up) and liver (bottom) stained with H&E from BALB/c mice (scale bar = 100 µm). Significant differences between groups were evaluated with ANOVA and Tukey–Kramer multiple comparison tests. * P < 0.05.

To confirm our in vitro findings, we examined the effect of chronic treatment with the drugs under study in two in vivo models of TNBC and CRC. CQ and P doses devoid of toxicity were selected based on the literature^6–8^. We observed that the combined treatment partially prevented the growth of TNBC, only for the 4T1 cells-based model (Fig. 3A). Interestingly, CQ + P decreased tumor growth (Fig. 3B–D) and metastasis development (Fig. 3E–G**, **Table 2) in both CRC evaluated models. In all the cases, individual treatments did not prevent significantly tumor growth.

**Table 2:** Metastasis count and splenomegaly identification in in vivo CRC models.

Organ	Control	CQ + P
CT26 injected mice		
Lung (nodes/mouse)	3 ± 0.29	1 ± 0.35*
Liver (nodes/mouse)	3.75 ± 0.34	2.5 ± 0.75
Splenomegaly	4/6	3/6
HCT116 injected mice		
Lung (nodes/mouse)	1.6 ± 0.22	0.6 ± 0.27*
Splenomegaly	3/5	1/5

Results are expressed as mean ± SEM for lung and liver metastasis. For spleen, the number of mice with splenomegaly/total number of animals is indicated.

*P < 0.05.

## Discussion

In oncology, drug repositioning explores the reuse of non-cancer drugs to treat malignant tumors and to facilitate patient access to new treatment options.

The individual effects of CQ and P have been studied in different cancer cell types both in vitro and in vivo. There is evidence indicating that CQ sensitizes cancer cells to chemotherapeutic drugs and radiation, although the precise mechanism is still unclear^6^. Retrospective studies associated the use of β-AR antagonists like P, with reduced tumor proliferation rates and decreased metastatic development^7^.

Therefore, we sought to evaluate whether CQ and P combination could be a good strategy to treat TNBC and CRC. First, we confirm that CQ and P decrease cells viability in a dose-dependent manner in different tumor models for these cancer types, and later we demonstrated that their combination is more effective than any individual treatment in all the tumor cells analyzed, with no effect on the MDCK non-tumor cell line. The same effect was previously observed by our group for the combination of P with metformin, another repositioned drug^5,9^. Also, we proved that CQ and P act synergically preventing tumor cell growth, and that continuous exposure to these drugs for a long period of time increased sensitivity. Similarly, it was reported that P inhibited the proliferation of liver cancer cells, and this inhibitory effect was enhanced by prolonged duration of treatment, increasing also apoptosis rate^10^.

The effect of CQ and P have been previously shown in other tumor models. It was reported that CQ and Hydroxychloroquine inhibited proliferation of human bladder cancer cells in a time and dose-dependent manner, also decreasing colony formation^11^. In our work, we showed that the combined treatment not only affected proliferation, but also significantly decreased cells clonogenic potential, although this effect seemed to be more evident for CRC cells. Likewise, we previously described that P, both alone or combined with metformin, significantly decreased clonogenicity and increased apoptosis in different TNBC and CRC cells^5,9^. Similarly, it was previously reported that CQ has an important effect on melanoma cell viability and apoptosis^12^. Accordingly, despite the fact that we observed an effect of chloroquine alone on the apoptosis levels of the HCT116 cell line, we report here that CQ + P combination increased apoptosis in comparison to control and individual treatments, suggesting propranolol could enhance the apoptotic effects described for chloroquine.

Both TNBC and CRC are highly metastatic types of tumors, which makes it important to find therapies able to prevent the formation of secondary nodes. To metastasize, cells need to migrate and invade tumor surroundings. We evaluated the potential effect of CQ, P and their combination to prevent TNBC and CRC cells in vitro, observing no effect for the treatment on TNBC 4T1 cells but a clear migration inhibition in different models of CRC. The effect of CQ on breast cancer cell migration was previously explored by other groups^13^. While we did not observe an in vitro effect on 4T1 cells, it was described an inhibition of in vivo migration for D2A1 TNBC cells in mouse mammary gland^14^. Despite this discrepancy, we observed a clear effect of CQ, P and particularly their combination on CRC cells migration, suggesting that the combined treatment could prevent metastasis development in this type of cancer. This migratory inhibition was previously described by us for P on CRC cells^5^ and it was also reported for CQ on MGC803 gastric cancer cells^15^.

Interestingly, the effects of CQ and P that we observed in vitro were conserved for CRC in vivo. Combined treatment of mice with CQ + P reduced tumor growth and prevented metastasis development in two different models of this disease. The effect of CQ and P separately on CT26 in vivo model was previously described^16,17^ but here we show the potential of combining these drugs on tumor growth and metastasis development with no signs of toxicity associated (Supplementary Fig. S1C). Indeed, CQ + P treatment also prevented the development of intestinal tumors in a chemically induced carcinogenesis assay (Supplementary Fig. S1D). According to our in vivo results, there is no clear benefit of this treatment on TNBC growth.

Altogether, our results indicate that CQ + P could be effective to treat CRC. It would be interesting to check the effect of this combination in other in vitro and in vivo models. It should be noted that, as far as we know, this is the first time that repositioned CQ + P combination was tested. Further investigations are necessary to explore the mechanisms implied in treatment effects.

## Supplementary Information

Supplementary Information.

Supplementary Legends.

Supplementary Figure S1.

## Data Availability

Datasets used and/or analysed during the current study are available from the corresponding author on reasonable request.
