# Supplementary material for: Potential effect of chloroquine and propranolol combination to treat colorectal and triple-negative breast cancers
Source: Sci Rep. 2023 May 16;13:7923. doi: 10.1038/s41598-023-34793-6 (PMC10188563; doi:10.1038/s41598-023-34793-6)
Supplement: Supplementary file 1 — Supplementary Information. [file 41598_2023_34793_MOESM1_ESM.docx]

**SUPPLEMENTARY DATA**

**
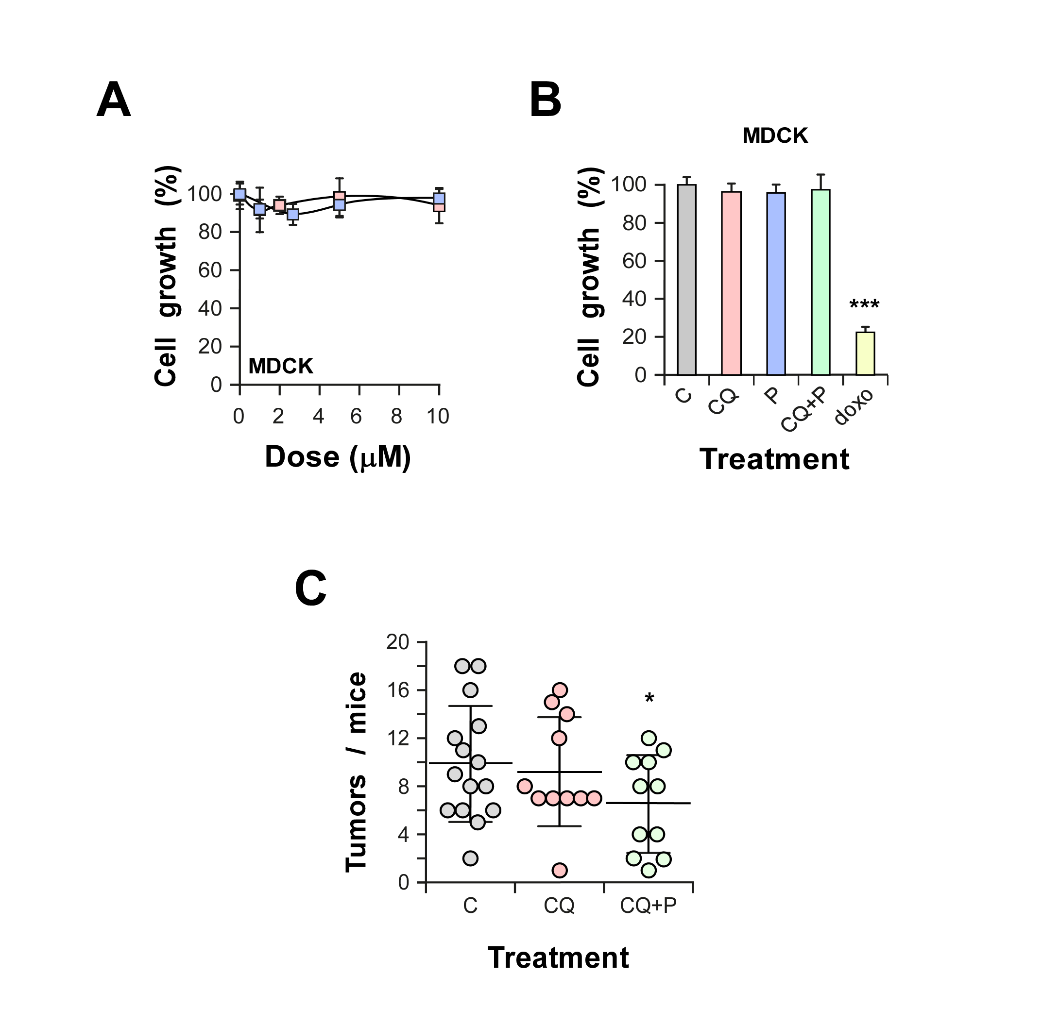
**

**Supplementary Figure S1:** (**A,B**) Analysis of the effect of CQ and/or Prop on the non-tumoral kidney-derived cell line MDCK. CQ 2.5 µM; Prop 2.5 µM; doxorubicin (doxo) 1 µM. (**C**) Chemically induced carcinogenic model. After carcinogenesis, mice intestines were collected and stained with methylene blue. The tumors were visualized under a stereoscopic microscope and quantified (n_CTRL_=15; n_CQ_= 11; n_CQ+P_=11). Significant differences between groups were evaluated with ANOVA and Tukey-Kramer multiple comparison tests. * P<0.05; ***P>0.001.

**SUPPLEMENTARY MATERIAL AND METHODS**

**Non-tumoral cell studies**

MDCK cells were kindly provided by Dra. Larocca. Cells were grown in DMEM media and treated as described before for tumor cell lines.

**Carcinogenesis model**

8-week-old BALB/c mice were intraperitoneally injected with azoxymethane (Santa Cruz Biotechnology) and then treated with sodium dextran sulfate (DSS; Santa Cruz Biotechnology) as described before^4^. Two months later the last round of DSS animals were randomly distributed in groups and treated as indicated before for four weeks. Then, mice were sacrificed, colon removed and tumors quantified after 0.5% methylene blue staining.
