# Supplementary material for: Potential effect of chloroquine and propranolol combination to treat colorectal and triple-negative breast cancers
Source: Sci Rep. 2023 May 16;13:7923. doi: 10.1038/s41598-023-34793-6 (PMC10188563; doi:10.1038/s41598-023-34793-6)
Supplement: Supplementary file 2 — Supplementary Legends. [file 41598_2023_34793_MOESM2_ESM.docx]

**Supplementary Figure 1: (A,B)** Effect of individual and combined treatments on the non-tumoral cell line MDCK. Doxorubicin (doxo) was carried out as a control. **(C)** Evolution of weight of BALB/C animals under the indicated treatments. The result shows a representative graft experiment. Essential weight changes were observed for every round. **(D)** Number of azoxymethane chemically induced tumors. Carcinogenesis was carried out as described before^5^.
