# Supplementary figures and images for: Potential effect of chloroquine and propranolol combination to treat colorectal and triple-negative breast cancers
Source: Sci Rep. 2023 May 16;13:7923. doi: 10.1038/s41598-023-34793-6 (PMC10188563; doi:10.1038/s41598-023-34793-6)

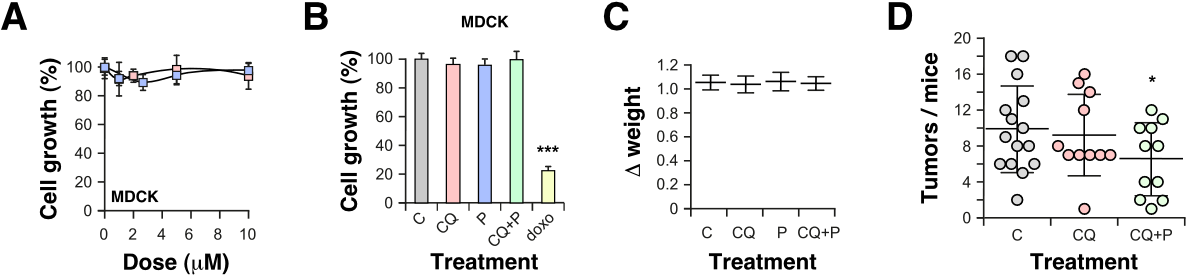

Supplement: Supplementary file 3 — Supplementary Figure S1. [file 41598_2023_34793_MOESM3_ESM.tif]
